# Individual Prediction of Optimal Treatment Allocation Between Electroconvulsive Therapy or Ketamine using the Personalized Advantage Index

**DOI:** 10.21203/rs.3.rs-3682009/v1

**Published:** 2023-11-30

**Authors:** Benjamin Wade, Ryan Pindale, Joan Camprodon, James Luccarelli, Shuang Li, Robert Meisner, Stephen Seiner, Michael Henry

**Affiliations:** Department of Psychiatry, Massachusetts General Hospital and Harvard Medical School, Boston, MA, USA; Department of Psychiatry, Massachusetts General Hospital and Harvard Medical School, Boston, MA, USA; Department of Psychiatry, Massachusetts General Hospital and Harvard Medical School, Boston, MA, USA; Department of Psychiatry, Massachusetts General Hospital and Harvard Medical School, Boston, MA, USA; Department of Psychiatry, McLean Hospital, Belmont, MA, USA; Department of Psychiatry, Massachusetts General Hospital and Harvard Medical School, Boston, MA, USA; Department of Psychiatry, McLean Hospital, Belmont, MA, USA; Department of Psychiatry, McLean Hospital, Belmont, MA, USA; Department of Psychiatry, Massachusetts General Hospital and Harvard Medical School, Boston, MA, USA

**Keywords:** depression, ketamine, electroconvulsive therapy, treatment selection, machine learning

## Abstract

**Introduction:**

Electroconvulsive therapy (ECT) and ketamine are two effective treatments for depression with similar efficacy; however, individual patient outcomes may be improved by models that predict optimal treatment assignment. Here, we adapt the Personalized Advantage Index (PAI) algorithm using machine learning to predict optimal treatment assignment between ECT and ketamine using medical record data from a large, naturalistic patient cohort. We hypothesized that patients who received a treatment predicted to be optimal would have significantly better outcomes following treatment compared to those who received a non-optimal treatment.

**Methods:**

Data on 2526 ECT and 235 mixed IV ketamine and esketamine patients from McLean Hospital was aggregated. Depressive symptoms were measured using the Quick Inventory of Depressive Symptomatology (QIDS) before and during acute treatment. Patients were matched between treatments on pretreatment QIDS, age, inpatient status, and psychotic symptoms using a 1:1 ratio yielding a sample of 470 patients (n=235 per treatment). Random forest models were trained and predicted differential patientwise minimum QIDS scores achieved during acute treatment (min-QIDS) scores for ECT and ketamine using pretreatment patient measures. Analysis of Shapley Additive exPlanations (SHAP) values identified predictors of differential outcomes between treatments.

**Results:**

Twenty-seven percent of patients with the largest PAI scores who received a treatment predicted optimal had significantly lower min-QIDS scores compared to those who received a non-optimal treatment (mean difference=1.6, t=2.38, q<0.05, Cohen’s D=0.36). Analysis of SHAP values identified prescriptive pretreatment measures.

**Conclusions:**

Patients assigned to a treatment predicted to be optimal had significantly better treatment outcomes. Our model identified pretreatment patient factors captured in medical records that can provide interpretable and actionable guidelines treatment selection.

## Introduction

Major depressive (MD) episodes affect an estimated 21 million adults in the United States annually [1]. Although numerous pharmaceutical, behavioral, and neuromodulatory treatments are available, only about one third of patients achieve remission following standard first line treatments [2] and after two or more antidepressant treatments, patients are classified as having treatment-resistant depression (TRD) [3]. This high rate of failed treatment response has heightened an interest in the development of more personalized medicine strategies [4] that might prospectively allocate patients to treatments best suited for them. Following this, numerous studies have investigated specific predictors or correlates of antidepressant treatment outcomes using neuroimaging [5–7], genetic [8–10], and clinical history measures [11–13]. While identification of treatment-responsive biomarkers for a given treatment is highly valuable, within-treatment studies do not provide clinicians with a direct means by which to differentially assign patients to an optimal treatment.

To address this limitation, DeRubeis et al. [14] introduced the Personalized Advantage Index (PAI). The PAI method is a means of identifying treatment-prescriptive biomarkers that predict differential treatment outcomes across two or more treatments [15]. Applied to a set of two or more treatments, the PAI approach provides a prediction of an optimal treatment as well as an expected magnitude of differential outcomes across each treatment, thus providing clinicians with an actionable metric for clinical decision making. The DeRubeis article applied the method to a cohort of patients with moderate to severe MD enrolled in a comparative clinical trial comparing cognitive behavioral therapy (CBT) to paroxetine. Clinical and demographic data was used to predict individual PAI scores and identify an optimal treatment for each patient. Patients who were randomly assigned to their predicted optimal treatment had significantly more reduced symptoms compared to those who had been randomly assigned to their predicted non-optimal treatment. Subsequent studies have applied the PAI method to identify prescriptive biomarkers of cognitive therapy versus interpersonal psychotherapy [15] and face-to-face CBT versus blended internet-based CBT [16]. To date, however, the PAI method has not been applied to predict treatment allocation for secondary lines of treatment commonly prescribed for patients with TRD, including electroconvulsive therapy (ECT) and ketamine.

ECT is a rapidly acting and highly effective treatment for TRD. Previous studies have reported that patients with psychotic features of depression and older patients are generally most responsive to ECT [17]. Ketamine is an N-methyl-d-aspartate receptor antagonist commonly used for anesthetic purposes. Subanesthetic doses of ketamine administered at 0.5 mg per kilogram body weight has more recently been used as an effective and rapidly acting treatment for MDD and TRD [18, 19]. Well-replicated predictors of antidepressant response to ketamine have included a higher body mass index and a positive family history of alcohol abuse [11, 12]. A recent open label, randomized, noninferiority trial compared antidepressant response rates between ECT (n = 170) and ketamine (n = 190; administered twice weekly for three weeks) in patients with TRD without psychosis and reported that ketamine was non-inferior to ECT [20].

In this study, we adapted and applied the PAI framework to a large, retrospective cohort of patients who underwent ECT or ketamine to treat TRD to generate individual predictions of patient outcomes for each treatment using pretreatment medical record and demographic measures. Following earlier studies using the PAI method, we hypothesized that patients who received a treatment predicted to be optimal for them would have significantly lower depressive symptoms following treatment compared to those who received the sub-optimal treatment.

## Materials and Methods

### Participants

Medical record data on 2671 patients who underwent ECT (n = 2526) or ketamine (n = 235) at McLean Hospital between 2011 and 2022 was aggregated from EPIC and mapped to medical records at McLean. Ketamine patients received either IV ketamine (n = 190) and esketamine (Spravato; n = 43) with the remaining 2 ketamine patients having unidentifiable IV or esketamine treatments. Patients were included in this study if they had a clinical diagnosis of depression (unipolar or bipolar). Diagnosis was made by the Referring psychiatrist or Psychiatric nurse practitioner and confirmed by the Consulting MD during clinical assessment. Patients were treated with either ECT or ketamine at the study site. No patients under 18 years of age were included. A diagnosis of psychosis was exclusionary for the ketamine cohort, however, a minority of ketamine patients endorsed symptoms of psychosis captured by the Behavior and Symptom Identification Scale (BASIS) psychosis [21]. Depressive symptoms were assessed using the Quick Inventory of Depressive Symptomatology (QIDS) Self Report Scale [22]. Patients were assessed over the acute phase of treatment, defined as the first 10 treatments for ECT and the first 8 treatments for ketamine. ECT patients completed the QIDS before the first treatment and after every 5th subsequent treatment. Ketamine patients completed the QIDS prior to each treatment. Patients were included in the cohort if the pre-treatment QIDS ≥ 10, indicating at least moderate depression severity. Treatment was provided as part of routine clinical care, as described below. This retrospective analysis of clinical records was approved by the Mass General Brigham IRB with a wavier of informed consent.

### Participant Matching

Earlier studies using the PAI method have used data on randomized controlled trials to avoid potential confounds arising from naturalistic settings in which patient characteristics inform treatment selection. Data used in this study is based on naturalistic patient assignment to either ECT or ketamine and, as such, patient symptom severity, proportion of inpatients, psychotic features of depression, and age, differed significantly between arms. To adjust for this, we used propensity score matching (PSM) [23] to match patients between treatments on pretreatment QIDS scores, inpatient status, severity of psychotic features captured by the 24-item BASIS psychosis subscale [21], and age using a 1:1 ratio, yielding a final sample of 470 patients (n = 235 per treatment). An outline of patient characteristics after matching is provided in Table 1. A summary of patient characteristics prior to matching is provided in Table S1.

### Electroconvulsive Therapy

ECT was provided using a Mecta Spectrum 5000Q instrument (Tualatin, OR) with individualized seizure threshold determination at the time of first treatment, as previously reported [24]. Subsequent treatments were delivered initially at 6x seizure threshold for right unilateral treatments, typically three times weekly. Electrode placement and ECT charge were then adjusted based on response by the treating psychiatrist [25], with details of the clinical treatment course previously reported. Methohexital was the default anesthetic, although etomidate, propofol, or ketamine anesthetic could be used at the discretion of the treating anesthesiologist. Muscle relaxation was provided by succinylcholine.

### Ketamine and Esketamine Treatment

IV ketamine and esketamine treatment were offered to patients with TRD defined by a history of two or more treatment failures with standard anti-depressants at adequate dosing and duration (as best could be determined in a naturalistic, clinical setting and in collaboration with their outpatient referring provider.) Patients with a history of psychosis, current substance use disorder, and relevant uncontrolled medical (i.e., arteriovenous malformation, uncontrolled HTN, aneurysmal disease) were deemed ineligible. Prior to initiation of treatment, each patient evaluated by an affiliated Internal Medicine physician or NP to obtain medical clearance. At each treatment, patients were evaluated and monitored by a ketamine-trained staff psychiatrist, and a ketamine-trained nurse. Patients were monitored with pulse oximetry, automated Blood Pressure monitoring and one:one nursing care in private, low-stimulation treatment rooms. An Anesthesia physician was available if needed.

Patients treated with I.V racemic ketamine began at a standard initial dose of 0.5 mg / kg administered over 40 minutes. Patients who failed do demonstrate clinically meaningful response by integrated subjective report, objective clinical assessment and evaluation of QIDS scores were advised to cease further treatment following treatment #3-#4. Dose was adjusted at the staff psychiatrist’s clinical discretion (but could not exceed 1.0 mg / kg) over the course of the treatment series according to clinical response. A full course of IV racemic ketamine was defined as 8 total treatments.

Esketamine was delivered in accordance with the 3-phase protocol with REMS monitoring mandated by the product label. All participants received training and practiced using the intranasal device before the first administration. Participants self-administered intranasal study drug at the clinical site under the direct supervision of the esketamine-trained nurse. Most participants received the first dose of 56 mg with the possibility of increasing the dose to 84mg contingent on patient tolerability to the index dose and according to the patient’s response. All participants were monitored at the clinic for to two hours following treatment.

During the treatments, other pharmacological and psychotherapeutic treatments were continued as part of the usual regimen. During and after the procedure, patients who experienced nausea could receive ondansetron. Metoprolol or Versed was available for blood pressure control. Criteria for discharge readiness included a return to baseline mental status, absence of gait disturbance and nausea, and normal blood pressure. Any administration required the patient to be discharged to the care of an adult escort.

### Clinical and Demographic Predictors

Predictors for our models were 112 demographic and pretreatment medical record measures including a treatment label (ketamine or ECT), the 24-item BASIS relationships, self-harm, emotional lability, psychosis, and substance abuse subscales [26], the Montreal Cognitive Assessment (MoCA) scale [27], indicator variables for medication history, and comorbid neurological, psychiatric, or general health diagnoses, race (White, Black or African American, Asian, or other) and ethnicity (Hispanic or Latino), age, and sex. A tabulation of predictor variables is given in Table S2.

MoCA and BASIS subscale scores were missing for a subset of patients and filled using imputation within treatment group. BASIS subscales were missing for roughly 2–3% of the ECT or ketamine group while 29% of MoCA scores were missing for the ketamine cohort. Missing MoCA scores and missing BASIS subscales were imputed within treatment classes. Missing MoCA scores, BASIS: psychosis and substance abuse scores were filled using the mode due to distributional skews. BASIS: emotional lability, relationship, and self-harm scores were imputed using mean due to the more normal or uniform distributions.

### Calculation of the Personalized Advantage Index

To calculate PAI scores, we trained a series of random forest regression (RFR) models to predict the minimum QIDS score achieved by patients over the acute course of treatment (min-QIDS), drawn from the 5th or 10th treatment for ECT patients and 2nd to 8th treatment for ketamine patients. All 112 pretreatment clinical and demographic variables described above were included as predictors. Predictions of min-QIDS scores were made using leave-one-out cross-validation (LOOCV) wherein models were trained on n-1 participants and the fitted model was used to predict min-QIDS in the n^th^ held-out participant. Each RFR model had 1000 underlying regression trees and was fit using 10-fold cross validation with nested feature selection and a grid search which were embedded sequentially in a secondary 10-fold cross validation applied to the training data. Feature selection proceeded in two steps: first, a near-zero-variance filter was used to remove variables in the training data that had only one unique value or a high ratio (95:5) of the most frequent value to second most frequent variable value. Second, remaining features were ordered based on their permutation-based importance scores [28] derived from a nested RFR model trained to predict min-QIDS in the training data. The number of features retained was the minimum of the number of features with non-zero importance scores or the upper 70% of the most important features. RFR parameters optimized in the nested grid search included *mtry* (the number of variables to consider split for each node split), *splitrule* (a function to evaluate the quality of each potential node split), *min.node.size* (the minimum number of samples in a node to allow a further split), and *n.filter* (the number of features to retain following feature selection). Random forest models were fit using the *ranger* package [29] and nested cross validation was implemented using the *nestedcv* package [30] in R version 4.3.0 [31].

As outlined in the DeRubeis article [32], two min-QIDS predictions were made for each held-out patient: one using the patient’s true treatment label and a counterfactual prediction in which the treatment label was switched to the treatment the patient did not receive. The prediction resulting in the lowest min-QIDS score was deemed the patient’s predicted optimal treatment while the prediction resulting in the larger min-QIDS score was deemed the patient’s non-optimal treatment. The magnitude of the difference between the optimal and non-optimal min-QIDS predictions is referred to as the patients predicted advantage: the PAI score. To test the hypothesis that patients who received their predicted optimal treatment would have lower min-QIDS scores following treatment, we compared distributions of min-QIDS scores between patients who received their predicted optimal treatments to those who did not using a two-sample, one-sided t-test.

In clinical practice, however, a patient with a PAI score near zero would not be expected to respond preferentially to one treatment over another; thus, treatment selection would be determined by other factors such as personal preference. Therefore, we examined differences in outcomes between patients who received optimal versus non-optimal treatments in the subset of patients with increasingly higher PAI values, from 0 to the maximum PAI score in steps of 0.1. T-tests across this range of PAI scores were adjusted for multiple comparisons using an FDR adjustment.

### Model Evaluation

A global RFR model was fit to the whole dataset to interpret the contributions of features and feature interactions in the prediction of min-QIDS. This model was trained using the same steps described above without the outer LOOCV loop. The performance of the global RFR model and the series of RFR models generating PAI scores were evaluated using the sum of squares formulation of the R^2^ (coefficient of determination) measure [33] which describes the fraction of explained variance in the min-QIDS measure captured by our models. The significance of the R^2^ scores was assessed using permutation tests with B = 1000 permutations of the entire modeling procedure in which the min-QIDS score was randomly reshuffled across patients at each iteration.

### Evaluation of Prognostic and Prescriptive Measures

Earlier studies applying the PAI method have distinguished between prognostic and prescriptive predictors. Here, prognostic predictors refer to baseline predictors that are indicative of an individual patient’s outcome following a treatment or a set of treatments, but do not indicate which treatment is expected to yield an optimal outcome. Prescriptive variables, in contrast, predict outcomes as a function of treatment type and can therefore inform optimal treatment selection [15].

The contributions of predictive features to model predictions at the group and individual patient level was evaluated using SHAP (SHapley Additive exPlanations) analysis [34] in the R-based *treeshap* package [35]. Through inspection of SHAP plots, we investigate three properties of our global RFR model: 1) *prognostic predictors* through evaluation of the overall importance of each feature in the prediction of min-QIDS and the directionality of important features with respect to predicted outcomes; 2) *prescriptive predictors* through inspection of SHAP interaction plots illustrating expected changes in outcomes that vary as a function of a predictor’s value and treatment type (ECT versus ketamine); and 3) decision paths for individual patients illustrating how observed values of their pretreatment clinical and demographic characteristics produced their predicted treatment outcome.

It is notable that earlier PAI implementations have investigated prescriptive predictors by inclusion of interaction terms between pretreatment predictors and treatment type. Tree-based regression/classification models such as RFRs, however, detect interaction effects through optimization of decision tree paths in which the influence of a given variable is conditioned on the value of preceding variables in the decision tree [36, 37]. Thus, we did not directly include interaction terms as model predictors but recovered them from the global RFR model through analysis of SHAP interaction values [35].

### Sensitivity Analyses

PSM is a useful method for adjust for known confounds in observational studies; however, it is not a perfect substitute for randomization as it cannot adjust for unmeasured confounds [38, 39]. Our main analysis uses PSM to match patients on pretreatment QIDS scores, psychotic symptoms from the BASIS-24 psychosis subscale, inpatient status, and age. This approach was taken because psychotic features of depression have been identified as predictive of response to ECT [40] and are often exclusionary for ketamine treatment [41]. The prevalence of psychotic features of depression differed significantly across treatment arms after matching on baseline QIDS. Similarly, the proportion of inpatients was higher in the ketamine group. To evaluate the sensitivity of our model’s performance to this choice of matching criteria, we repeated our analysis using several subsets of our matching criteria: 1) matching only on baseline QIDS; 2) matching on baseline QIDS and patient age; and 3) matching on baseline QIDS, age, and inpatient status.

### Subgroup Analyses

Ketamine patients were a mixture of IV ketamine and esketamine (Spravato). We repeated the above analyses using subsets of patients who received IV ketamine or esketamine. ECT patients were matched to each ketamine group using the same PSM matching procedures described previously.

### Power Analyses

We conducted power analyses for t-tests to identify the minimum effect size detectable given each sample size for the primary analysis and the ketamine subgroup analyses. Power was reported for two-sample, one-sided t-tests with a significance level of 0.05 with 80% power to detect a significant effect.

### Comparison of Treatment Arms

Patient characteristics reported in Table 1 were compared between treatment arms using t-tests and Chi-squared tests where appropriate.

## Results

### Cohort Characteristics

Demographic and clinical measures for the matched sample used for the main analysis are outlined in Table 1. Matching resulted in a sample of n = 470 patients (n = 235 per treatment arm). The mean baseline QIDS scores were 17.51±3.37 and 17.73±3.50 for the ECT and ketamine groups, respectively. Min-QIDS scores were 11.05±4.40 for the ECT and 10.77±4.71 for the ketamine cohorts. Neither baseline nor min-QIDS scores differed between cohorts. Average patient age was 43.64±16.37 and 42.61±16.34 years for the ECT and ketamine groups, respectively, and did not differ significantly. Patient sex did not significantly differ between groups with 131 (55.7%) females in the ECT cohort and 146 (62.1%) females in the ketamine cohort. The prevalence of bipolar disorder was significantly higher in the ECT cohort. Meanwhile, there was a higher frequency of comorbid diagnosis of anxiety, alcohol dependence, overall number of comorbid diagnoses, and number of medications taken prior to treatment in the ketamine cohort. Baseline and min-QIDS scores did not differ between the IV ketamine and esketamine cohorts. Details of the matched IV ketamine and esketamine cohorts are provided in Supplementary Tables 3 and 4, respectively.

### Personalized Advantage Index

**Figure 1:**
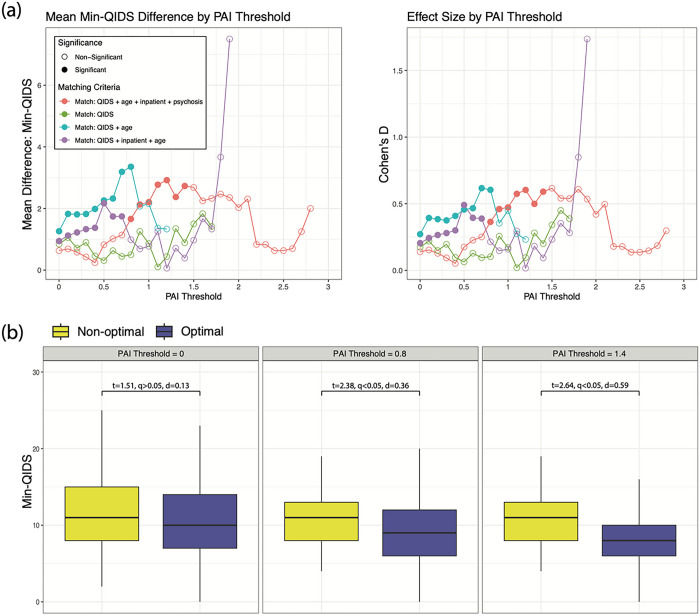
Panel (a, left) illustrates the mean difference in min-QIDS scores (y-axis) between patients who received optimal versus non-optimal treatments as a function of PAI thresholds (x-axis) ranging from 0 to the maximum PAI score in steps of 0.1. Filled-in points indicate that a significant difference in min-QIDS scores was observed after adjusting for multiple comparisons while empty points indicate non-significant differences. Separate lines are provided for each set of matching criteria. The red line is the primary model used in this analysis with patients matched on pretreatment QIDS, age, inpatient status, and psychotic symptoms. The additional lines report differences observed with our sensitivity analysis. Panel (a, right) reports the Cohen’s D effect size difference in min-QIDS scores between patients who received optimal versus non-optimal treatments. Last, panel (b) shows boxplots of distributions of min-QIDS scores between patients who received optimal and non-optimal treatments for several PAI scores in our primary model: PAI = 0, the entire sample; PAI = 0.8, the first threshold at which a significant between-group difference was observed; and PAI = 1.4 the final threshold at which a significant difference was observed.

The global RFR model predicted min-QIDS significantly above chance (mean R^2^ = 0.16, p < 0.001). ECT was predicted to be optimal for 62 (26%) of patients who received it while ketamine was predicted to be optimal for 173 (73%) of patients who received it. The average min-QIDS score for patients who received their optimal treatment (n = 235, 50%) was 10.59 while the average min-QIDS score for those who received their non-optimal treatment was 11.22. Across the whole of the sample, difference in min-QIDS scores was not significantly different between patients who received an optimal versus non-optimal treatment (p > 0.05). At a PAI threshold of 0.8, resulting in a subset of 129 patients (27.4% of patients, n = 75 ECT and n = 54 ketamine), patients who received their optimal treatment had a significantly lower min-QIDS score compared to those who received a non-optimal treatment (mean difference = 1.6, t = 2.38, q < 0.05, Cohen’s D = 0.36). Higher PAI thresholds resulted in significant effect sizes up to 0.59. Figure 1 illustrates mean differences in min-QIDS scores and effect sizes between patients who received optimal versus non-optimal treatments across a range of PAI thresholds for our main model and sensitivity analysis models. Power analysis revealed that the sample was powered to identify a small Cohen’s D effect size of d = 0.22.

### Prognostic Predictors

**Figure 2:**
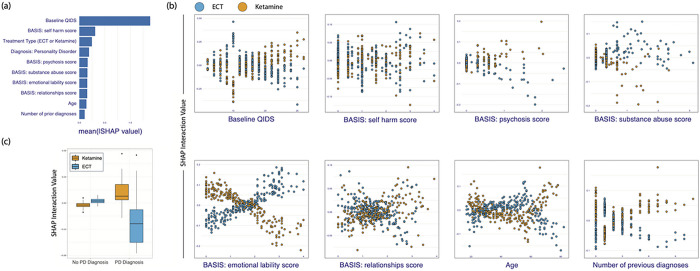
Panel (a) shows SHAP variable importance scores for the 10 most predictive pretreatment measures in our global model. Panel (b) illustrates SHAP interaction plots for continuous predictors. The y-axis shows SHAP interaction values where values above zero indicate an expectation of higher min-QIDS scores while values below zero indicate a lower expected min-QIDS score. Each point represents a patient and is color coded by treatment. The x-axis is the observed range of the predictor value. Panel (c) illustrates SHAP interaction values for the binary personality disorder variable.

**Figure 3:**
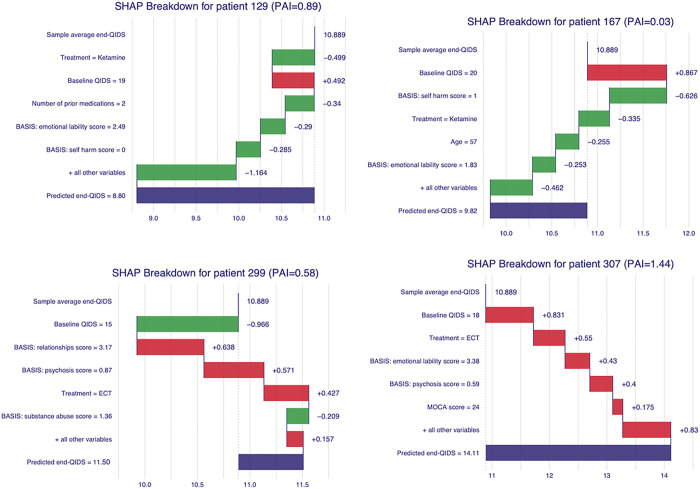
SHAP waterfall plots illustrate the contribution of individual predictors to the final prediction of individual patient min-QIDS scores for a random selection of four patients. The “Sample average min-QIDS” reflects the average min-QIDS score across the whole patient cohort. Each predictor is then represented as a horizontal bar which sequentially increase or decrease from the model’s prediction of min-QIDS for the given patient. Predictors that increase the model’s prediction of min-QIDS are filled in red while those that decrease its prediction are filled in green. Values for each patient’s predictor are given on the y-axis of each plot by the predictor’s name. The model’s final prediction is given in the last row as “Predicted min-QIDS”.

In descending order, the most important variables in the overall prediction of min-QIDS were pretreatment QIDS, BASIS: self-harm, treatment type, diagnosis of a personality disorder, BASIS: psychosis score, BASIS: substance abuse score, BASIS: emotional lability score, BASIS: relationships score, age, and the number of pre-existing diagnoses (neurological, psychiatric, general health). Figure 2(a) illustrates the overall feature importance scores for the top 10 most predictive features. SHAP waterfall plots illustrate the contributions of individual features to the prediction of individual patient outcomes for a selection of four patients in Fig. 3, illustrating how this model might be used to inform clinical decision-making.

### Prescriptive Predictors

Analysis of SHAP interaction plots indicated that min-QIDS predictions varied as a function of treatment type for several important predictor variables. Patients with pretreatment QIDS scores between 11 to 18 (moderate to severe depression) were expected to have marginally better outcomes with ketamine over ECT while patients with QIDS scores above 18 (severe to very severe depression range) were expected to have more reduced symptoms with ECT. Similarly, lower BASIS: self-harm scores predicted preferential outcomes for ketamine while higher scores predicted better outcomes for ECT. Diagnosis of a personality disorder predicted favorable outcomes for ECT while patients without a personality disorder were predicted to have marginally better outcomes with ketamine. Higher BASIS psychosis scores predicted better outcomes with ECT. Higher BASIS substance abuse scores generally predicted better outcomes with ketamine over ECT. Lower BASIS emotional lability scores predicted better outcomes with ECT while higher scores predicted favorable outcomes with ketamine. Patients between the ages of roughly 40 to 60 were anticipated to have favorable outcomes with ketamine while older patients over 60 were predicted to have more reduced symptoms with ECT. Last, patients with 3 or more comorbid diagnoses were expected to have better outcomes with ECT. Figure 2(b–c) illustrates SHAP interaction plots highlighting our model’s expectations for individual patient outcomes as a function of pretreatment predictors and treatment.

### Sensitivity Analyses

Matching on alternative subsets of variables yielded differing ranges of PAI values. Matching on baseline QIDS and age resulted in significantly lower min-QIDS scores in patients who received an optimal treatment (mean difference = 1.26, t = 2.94, q < 0.05, Cohen’s D = 0.27) without PAI thresholding. Similarly, matching on baseline QIDS, age, and inpatient status resulted in significantly lower min-QIDS scores in patients who received an optimal versus non-optimal treatment (mean difference = 0.94, t = 2.22, q < 0.05, Cohen’s D = 0.20); see Fig. 1(a).

### Subgroup Findings

Models using matched data for the IV ketamine (R^2^ = 0.12, p < 0.001) and esketamine (R^2^ = 0.17, p < 0.001) cohorts predicted min-QIDS significantly above chance. No significant differences in min-QIDS scores were observed between patients who received an optimal predicted versus non-optimal predicted treatment were observed in the subsamples matched for IV ketamine or esketamine cohorts. Prior to adjusting for multiple comparisons, however, significant differences were observed in the IV ketamine cohort when patients were matched on baseline QIDS and age as well as when patients were matched on baseline QIDS, age, and inpatient status; see **Supplementary Fig. 1**. Power analyses revealed that the IV ketamine cohort was powered to detect a small effect size of d = 0.28 while the esketamine cohort was powered to detect a medium effect size of d = 0.55.

Analysis of SHAP values showed that unique and overlapping predictors were important for models restricted to IV ketamine and esketamine cohorts. Prescriptive predictors generally followed the same patterns observed in the primary analysis in each ketamine subgroup, though several differences were observed. In IV ketamine, pretreatment QIDS, and self-harm symptoms were not differentially associated with treatment outcomes. Higher severity of psychotic symptoms favored outcomes for IV ketamine over ECT while symptoms of emotional lability and patient age followed the same patterns as the primary analysis.

In the esketamine cohort, higher pretreatment QIDS scores predicted better outcomes for ECT over esketamine while more severe BASIS: self-harm scores predicted better outcomes with esketamine over ECT. Emotional lability and age were not differential predictors of outcomes across treatments. SHAP interaction plots for the subgroup analyses are provided in **Supplementary Figs. 2–3.**

## Discussion

Prediction of optimal treatment allocation for individual patients is a central research aim in psychiatry. We developed a machine-learning adaptation of the Personalized Advantage Index approach originally developed by DeRubeis[32] to predict optimal treatment allocation between ECT and ketamine for individual patients using pretreatment measures of clinical records and demographic data. In this observational study, we matched patients on baseline depression severity, age, inpatient status, and severity of psychotic symptoms to more closely mimic what would be observed in a clinical trial. In the matched sample, treatment efficacy was equal which echoes a recent study confirming the noninferiority of ketamine to ECT as an antidepressant treatment for non-psychotic depression [20]. No significant differences in min-QIDS scores were observed between patients who received optimal versus non-optimal treatments when we compared all patients. However, this is somewhat expected as a large proportion of patients were predicted to have only marginal differences in outcomes between treatments as reflected by a small PAI score. In clinical decision making, treatment recommendations from this system would likely be made for patients with large differences in expected treatment outcomes. Conversely, treatment choices for patients with smaller PAI scores would likely be guided more by accessibility or personal preference [32]. Following this expectation, the 27% of patients with the largest PAI scores exhibited significant differences in min-QIDS scores with those assigned to an optimal treatment having average min-QIDS scores 1.6 to 2.9 points lower than those assigned to a non-optimal treatment, constituting small to medium effect size differences. Notably, however, a meaningful change threshold on the QIDS scale has been reported to be 3.5 [42], which is larger than differences detected in this study.

We evaluated results when alternative matching criteria were set as a sensitivity analysis. We noted that matching only on baseline QIDS scores resulted in no differences in outcomes, suggesting that adjusting for known predictors of outcomes in ketamine and ECT was needed to yield meaningful predictions. When we adjusted for only baseline QIDS and age, differences in min-QIDS scores were detected across the entire sample and required no PAI thresholding.

To our knowledge, this is the first application of the PAI method to predict treatment allocation outside of cognitive behavioral therapy, psychotherapy, or selective serotonin reuptake inhibitors (SSRIs) [15, 16, 43, 44]. An alternative strategy to predict individual likelihoods of treatment response to SSRIs, SNRI, bupropion, and mirtazapine treatments using electronic health record data similar to this study was recently developed by Sheu et al. [39].

There is an urgent need to optimize antidepressant treatment selection, particularly for patients suffering from TRD, which is associated with extended and costly inpatient care [46, 47]. Ketamine and ECT are two rapidly acting treatments for TRD with comparable efficacy in non-psychotic depression [20]. Patients may have personal preferences in selecting ECT or ketamine which may be informed by factors including, for instance, that ECT requires general anesthesia and has been linked with transient memory impairment [48]. Ketamine, however, has lability for abuse [20, 49] and is commonly not indicated for patients with psychotic features of depression. For patients with a negligible predicted difference in outcomes between these treatments, treatment selection may be informed by weighing these factors. Patients with a large differential in predicted outcomes, however, may also factor into their decision the expected difference in outcomes using this method.

### Treatment prescriptive factors and alignment with existing studies

Several factors informed preferential outcomes between ECT and ketamine in this observational sample; we discuss several of these below. Our models predicted better outcomes for patients with moderate to severe depression who used ketamine. Patients with severe to very severe depression, however, were expected to recover more with ECT. Pretreatment symptom severity is a commonly reported predictor of subsequent symptom change for both ECT and ketamine with more severe symptoms generally predicting poorer outcomes [50–52]. Relatedly, more severe self-harm was an indicator of preferential outcomes with ECT. Previous studies have reported that ECT reduces suicidal ideation [53, 54] and attempts [55], however, little has been reported about self-harm as a predictor of ECT response. An earlier report found that a prior history of suicide attempts predicted poorer outcomes for ketamine [12], in line with our current findings. Our model also predicted significantly higher efficacy of ECT for patients with a comorbid personality disorder. Previous studies have found that ECT is equally effective for depressed patients with comorbid borderline personality disorder (BPD) [56, 57]. Evidence is more limited for ketamine, however, a recent pilot study reported that ketamine reduced symptoms of BPD significantly compared to midazolam [58].

The BASIS scale captures several symptoms of psychosis including hallucinations and delusions. Our model predicted that patients with more severe psychotic symptoms would have better outcomes with ECT versus ketamine. Patients with psychotic features of depression are widely reported to respond well to ECT [40, 59] while psychotic symptoms are often exclusionary for treatment with ketamine [20]. Notably, a full diagnosis of psychosis was exclusionary for ketamine patients included in this study, however, some ketamine patients did endorse symptoms of psychosis captured by the BASIS scale which was not exclusionary.

Substance abuse is commonly associated with poor antidepressant treatment response [60]. Here, our model predicted favorable antidepressant outcomes for patients with more severe substance abuse who were treated with ketamine compared to ECT. This finding aligns with earlier reports that comorbid alcohol and drug abuse adversely affect antidepressant response to ECT [61] while ketamine is reportedly more effective for patients who have a first-degree relative with alcohol use disorder [12]. Moreover, ketamine has been reported to facilitate abstinence across multiple substances of abuse [62].

### Evaluation of ketamine subgroups

Because our primary analysis included ketamine patients who received either IV ketamine or esketamine, we conducted subgroup analyses matching ECT patients to IV ketamine and esketamine cohorts, separately. Differences in min-QIDS scores were seen between patients who received an optimal versus non-optimal treatment in the IV ketamine subgroup when patients were matched on baseline QIDS and age or baseline QIDS, age, and inpatient status; however, these results did not survive adjustment for multiple comparisons. We also did not observe differences in min-QIDS in the esketamine subgroup. Notably, neither baseline nor min-QIDS scores differed between patients who received IV ketamine or esketamine. The absence of robust differences in our subgroup analyses may be a function of reduced statistical power in with fewer observations and diminishing power at higher PAI thresholds which further reduce the number of observations used in each t-test.

Preclinical research suggests that (S)- and (R)-ketamine operate on differing mechanisms [63], however there is little literature comparing the antidepressant effects of racemic IV ketamine to esketamine. A recent study comparing IV ketamine to intranasal esketamine reported similar efficacy for both treatments [64], echoing observations in our cohort. In our subgroup analyses, there was a mixture of overlapping and unique predictors important in predicting min-QIDS scores. Specific prescriptive measures generally followed similar patterns in subgroups as in the primary analysis where IV ketamine and esketamine were mixed, though there were some notable differences. Specifically, in the IV ketamine subgroup matched to ECT, patient age and severity of psychotic symptoms did not indicate preferential outcomes between treatments as with the mixed group analysis and diagnosis of a personality disorder was associated with better outcomes in the IV ketamine group. Additionally, unlike the mixed group, the subgroup analysis of esketamine and ECT did not identify age and emotional lability as differential predictors of outcome while elevated self-harm symptoms favored esketamine. These differences, however, must be weighed against the observation that subgroup analyses were underpowered to detect differences in min-QIDS between optimal versus non-optimal treatment groups. Thus, additional studies are needed to resolve these potential differences in prescriptive markers.

### Modifications of the PAI method

It is notable that our approach differs slightly from previous implementations of the PAI method. As noted by Huibers et al. [15], earlier uses of the PAI method performed feature selection outside of cross validation, on the whole data set, which is likely to introduce information leakage bias [33] and inflate model performance. Here, we predicted patient outcomes using random forest regression models which are a class of embedded methods, meaning that feature selection is integrated into the model’s training [37, 65]. We used nested cross validation to tune model parameters and identify optimal subsets of predictors to avoid information leakage.

### Limitations

There are several important limitations to consider when interpreting these findings. Most PAI studies have had the advantage of using randomized clinical trial data which effectively minimizes the potential for introducing confounder bias. This study has used observational data and subsequent PSM to account for known confounds; however, PSM is unable to account for unknown/unmeasured confounds that may influence either naturalistic treatment allocation or subsequent outcomes [38, 39]. To partially address this limitation, matched patients on expected confounds including pretreatment symptom severity, age, inpatient status, and severity of psychotic symptoms. Using sensitivity analyses, we explored the results of our models if matching criteria were altered and generally observed that, after thresholding PAI scores, patients who received a treatment predicted optimal had significantly better outcomes. However, this was not true when matching only on baseline symptoms. Ketamine patients in our primary analysis were a mixture of IV ketamine and esketamine with a minority of patients missing clear records for IV ketamine versus esketamine. However, pre- and post-treatment symptom severity did not differ between ketamine subgroups and subgroup analysis supported similar findings in the larger IV ketamine cohort as the primary analysis. Moreover, barring a few exceptions, prescriptive predictors were generally similar in directionality in this subgroup analysis. Differences between the primary analysis and subgroup analysis may reflect reductions in overall statistical power. Another notable consideration is that the expected differences in min-QIDS scores between optimal and non-optimal treatment cohorts were 1.6 to 2.9, on average. A clinically significant change on the QIDS-SR scale, however, has been reported to be 3.5 [42]. Despite this, our findings support that allocation of a predicted optimal treatment results in significant symptom reductions ranging from small to medium effect sizes. Additional limitations derive from the retrospective observational nature of the study. For instance, study inclusion required the patient to be able to complete baseline severity measures, which may exclude the most severely ill patients who were unable to complete these assessments. Furthermore, diagnosis was determined by the treating clinician rather than by structured interview, which may hinder comparisons with prospective trials but matches ordinary clinical practice. Additionally, we cannot control for the effects of concomitant medication changes or psychotherapy treatment that may have occurred during the study period.

## Conclusions

Predicting which antidepressant treatment will elicit the most robust response from an individual patient is of the utmost importance. In this study, we adapted the PAI method to predict optimal treatment allocation between two equally effective rapidly acting treatments for TRD: ECT and ketamine. As hypothesized, patients who received a treatment predicted optimal had significantly better treatment outcomes reflecting small to medium effect size differences. Importantly, these models were constructed using commonly acquired and inexpensive demographic and medical record data. Precision medicine methods such as this have the potential to provide actionable predictions for both patients and clinicians in the selection of treatments and their use should be expanded to include additional treatment modalities.

## Figures and Tables

**Table 1:** Clinical and demographic characteristics of matched sample

Variable	ECT	Ketamine
N	235	235
Female sex, N (%)	131 (55.7)	146 (62.1)
Age years, Mean (SD)	43.64 (16.37)	42.61 (16.34)
Inpatient, N (%)	206 (87.7)	206 (87.7)
IV ketamine/Esketamine, N (%)	--	190 (80.8) / 43 (18.2)
**Race/Ethnicity**		
Hispanic, N (%)	6 (2.6)	5 (2.1)
Asian, N (%)	7 (3.0)	9 (3.8)
Black, N (%)	5 (2.1)	1 (0.4)
White, N (%)	208 (88.5)	205 (87.2)
Other, N (%)	7 (3.0)	16 (6.8)
**Psychiatric Symptoms/Diagnoses**		
Bipolar disorder, N (%)*	58 (24.7)	29 (12.3)
Post-traumatic stress disorder, N (%)	15 (6.4)	27 (11.5)
Anxiety, N (%)*	33 (14.0)	73 (31.1)
Insomnia, N (%)*	2 (0.9)	15 (6.4)
Personality disorder, N (%)	18 (7.7)	21 (8.9)
Eating disorder, N (%)	5 (2.1)	14 (6.0)
BASIS: relationships, Mean (SD)	1.45 (0.70)	1.51 (0.83)
BASIS: self-harm, Mean (SD)	1.00 (1.03)	1.06 (1.05)
BASIS: emotional lability, Mean (SD)*	1.63 (0.98)	1.42 (1.00)
BASIS: psychosis score, Mean (SD)	0.17 (0.43)	0.18 (0.37)
BASIS: substance abuse, Mean (SD)*	0.41 (0.68)	0.20 (0.40)
**Neurological Symptoms/Diagnoses**		
MoCA, Mean (SD)	26.34 (2.57)	26.66 (1.94)
Epilepsy, N (%)*	15 (6.4)	5 (2.1)
Concussion/brain injury, N (%)	3 (1.3)	5 (2.1)
Autism, N (%)	2 (0.9)	1 (0.4)
Migraines, N (%)	8 (3.4)	11 (4.7)
**General Health**		
Obesity, N (%)	11 (4.7)	16 (6.8)
Alcohol dependence, N (%)*	30 (12.8)	50 (21.3)
Nicotine dependence, N (%)	8 (3.4)	12 (5.1)
Cannabis dependence, N (%)	11 (4.7)	5 (2.1)
Opioid dependence, N (%)	2 (0.9)	0 (0.0)
Psychoactive dependence, N (%)	2 (0.9)	0 (0.0)
Back pain, N (%)	4 (1.7)	7 (3.0)
Diabetes type 1, N (%)	0 (0.0)	1 (0.4)
Diabetes type 2, N (%)	3 (1.3)	7 (3.0)
Number of prior diagnoses, Mean (SD)*	1.80 (1.75)	2.18 (2.21)
**Medication History Within 60 Days Before Treatment**		
Number of prior medications, Mean (SD)*	0.83 (1.67)	1.56 (2.21)
Mirtazapine, N (%)*	1 (0.4)	8 (3.4)
Duloxetine, N (%)	6 (2.6)	9 (3.8)
Venlafaxine, N (%)	5 (2.1)	10 (4.3)
Vortioxetine, N (%)	0 (0.0)	3 (1.3)
Fluvoxamine, N (%)	0 (0.0)	3 (1.3)
Selegiline, N (%)	1 (0.4)	4 (1.7)
Clomipramine, N (%)	0 (0.0)	2 (0.9)
Paroxetine, N (%)	1 (0.4)	1 (0.4)
Nortriptyline, N (%)	2 (0.9)	1 (0.4)
Fluoxetine, N (%)	4 (1.7)	5 (2.1)
Sertraline, N (%)	6 (2.6)	4 (1.7)
Citalopram, N (%)	4 (1.7)	12 (5.1)
Escitalopram, N (%)*	2 (0.9)	11 (4.7)
Seroquel, N (%)	17 (7.2)	16 (6.8)
Abilify, N (%)	10 (4.3)	13 (5.5)
Latuda, N (%)	8 (3.4)	4 (1.7)
Cariprazine, N (%)	1 (0.4)	3 (1.3)
Haldol, N (%)	1 (0.4)	0 (0.0)
Olanzapine, N (%)	4 (1.7)	7 (3.0)
Risperidone, N (%)	5 (2.1)	3 (1.3)
Perphenazine, N (%)	2 (0.9)	0 (0.0)
Chlorpromazine, N (%)	1 (0.4)	1 (0.4)
Brexpiprazole, N (%)	1 (0.4)	1 (0.4)
Ziprasidone, N (%)	1 (0.4)	0 (0.0)
Ativan, N (%)	27 (11.5)	26 (11.1)
Diazepam, N (%)	6 (2.6)	3 (1.3)
Clonazepam, N (%)	12 (5.1)	13 (5.5)
Chlordiazepoxide, N (%)	2 (0.9)	1 (0.4)
Alprazolam, N (%)	2 (0.9)	5 (2.1)
Buspirone, N (%)	2 (0.9)	5 (2.1)
Lamotrigine, N (%)	13 (5.5)	12 (5.1)
Depakote, N (%)	2 (0.9)	2 (0.9)
Gabapentin, N (%)	8 (3.4)	9 (3.8)
Pregabalin, N (%)	0 (0.0)	5 (2.1)
Topiramate, N (%)	4 (1.7)	1 (0.4)
Oxcarbazepine, N (%)	1 (0.4)	0 (0.0)
Methylphenidate, N (%)*	0 (0.0)	6 (2.6)
Lisdexamfetamine, N (%)	0 (0.0)	5 (2.1)
Modafinil, N (%)	0 (0.0)	4 (1.7)
Dextroamphetamine, N (%)	2 (0.9)	7 (3.0)
Ketamine, N (%)*	2 (0.9)	96 (40.9)
Esketamine, N (%)*	0 (0.0)	19 (8.1)
Metoprolol, N (%)	3 (1.3)	9 (3.8)
Atenolol, N (%)	3 (1.3)	1 (0.4)
Propranolol, N (%)	2 (0.9)	5 (2.1)
Zolpidem, N (%)	4 (1.7)	2 (0.9)
Zaleplon, N (%)	0 (0.0)	1 (0.4)
Lithium, N (%)	9 (3.8)	6 (2.6)
Prazosin, N (%)	6 (2.6)	3 (1.3)
Benztropine, N (%)	3 (1.3)	0 (0.0)
**Depression Severity**		
QIDS baseline, Mean (SD)	17.51 (3.37)	17.73 (3.50)
Minimum Treatment QIDS, Mean (SD)	11.05 (4.40)	10.77 (4.71)

Abbreviations: ECT: Electroconvulsive therapy; BASIS: Behavior and Symptom Identification Scale; QIDS: Quick inventory of depressive symptomatology; MoCA: Montreal Cognitive Assessment

* Significant between-treatment difference at *α* 0.05 level.

## Data Availability

The dataset used for this study is not publicly available due to patient privacy concerns but may be made available from the corresponding author on request.
